# Relations among Temperament, Self-regulatory Strategies and Gender in Predicting Delay of Gratification

**DOI:** 10.3389/fpsyg.2017.01925

**Published:** 2017-11-01

**Authors:** Fang Hong, Stacey N. Doan, Angelica Lopez, Gary W. Evans

**Affiliations:** ^1^Department of Psychological and Brain Sciences, Boston University, Boston, MA, United States; ^2^Applied Mind and Health Laboratory, Department of Psychology, Claremont McKenna College, Claremont, CA, United States; ^3^Department of Design and Environmental Analysis and Department of Human Development, Cornell University, Ithaca, NY, United States

**Keywords:** temperament, self-regulation, strategy, delay of gratification, gender

## Abstract

Self-regulation is associated with many positive outcomes, but there is limited information about individual difference regarding children’s spontaneous use of strategies to self-regulate and the relative success of those strategies. In the current study, we examined whether temperament and gender are associated with self-regulation and explored the types of spontaneous strategies children use during Mischel’s delay of gratification protocol. In addition, we investigated whether spontaneous strategy use during the task could moderate the effects of temperament on self-regulation and whether temperament would mediate the effect of gender on self-regulation. Participants were 349 9-year-olds (182 boys, *M*_age_ = 9.18, *SD* = 1.17). Mothers reported on children’s temperament and the Delay of Gratification task was used to assess self-regulation. Both temperament and child’s gender were significantly associated with children’s delay time. Girls were able to delay longer than boys, and children scoring high on activity level were less able to delay. Activity level also mediated the relationship between gender and delay time. Finally, we found an interaction effect between activity level and certain strategies in relation to self-regulatory behavior.

## Introduction

Self-regulation is conceptualized as “controlled, cognitive monitoring of the actions and steps required to obtain a goal, or to bring about a desired response from the environment” (Blair, 2003, p. 1). The ability to self-regulate one’s behaviors at a young age is associated with a host of subsequent positive outcomes, including social competence, academic success, and mental and physical health (e.g., Shoda et al., 1990; Kochanska et al., 2000; Calkins and Fox, 2002; Kochanska and Knaack, 2003; Baumeister and Vohs, 2004; Blair and Razza, 2007; Blair, 2010; Vohs and Baumeister, 2011; Mischel, 2014; Blair and Raver, 2015). Delay of gratification, the postponement of immediate gratification in pursuit of more attractive but delayed rewards, is central to effective self-regulation and adaptive social and behavioral development (Mischel et al., 1989; Mischel, 2014). Effective delay gratification in childhood predicts higher self-regulation in pursuit of goals as an adult (Moffitt et al., 2011). On the other hand, poor performance in a delay of gratification (DoG) task during childhood is associated with delinquency and socially irresponsible behaviors, and predicts poor academic performance and compromised social and coping skills (Mischel, 1961, 2014; Mischel et al., 1988).

Past research has explored both biological underpinnings, such as temperament and personality, and higher level strategies or skills that may contribute to individual differences in responses to the DoG task (e.g., Mischel and Ebbesen, 1970; Mischel and Mischel, 1983; Peake et al., 2002; Mittal et al., 2013). Recent studies in cognitive neuroscience suggest that effective self-regulation likely recruits top-down processes that modulate lower level emotional processes. For example, frontostriatal white matter integrity explained variance in current and future delay of gratification ability (Achterberg et al., 2016). Delay of gratification is associated with individual differences in white matter connectivity in the dorsal prefrontal cortex (e.g., Drobetz et al., 2014; Latzman et al., 2015) and was also associated with limbic system (e.g., reviewed in Zayas et al., 2014). In addition, children’ ability to delay gratification also predict their biases in frontostriatal circuitries that integrate motivational and control processes 40 years later (Casey et al., 2011). However, few studies have examined potential interactions between temperament and deliberate strategy use in the prediction of self-regulation (specifically, delay of gratification) during the DoG task. Moreover, previous research found that boys and girls differed in temperament and self-regulation (e.g., Maccoby and Jacklin, 1974; Else-Quest et al., 2006). Yet to date there appears to have been no research determining whether temperament would mediate the relationship between child’s gender and self-regulation. Finally, most studies on strategy use have looked at preschool children, where cognitive capacities may limit the type of strategies available to them. The current study attempts to address these gaps in the literature.

### Temperament and Self-regulation

Temperament has been described as “simple, non-motivational, non-cognitive stylistic characteristics that represent meaningful ways of describing individual differences between people” (Rutter, 1987, p. 447); these characteristics appear early in childhood and are relatively stable. Temperament largely determines how children experience and interpret their world, as well as how they react to life experiences (Kristal, 2005). Temperament is commonly assessed using the three main dimensions proposed by Buss and Plomin (1975): activity (e.g., quantity of motor activity), sociability (e.g., closeness to others), and emotionality (e.g., intensity of emotion). They described activity as the tendency to be restless or energetic and included the sub-scales tempo and vigor. Sociability is the tendency to prefer being in the presence of others rather than being alone and seeking out social interaction. Emotionality is the tendency to become upset easily.

In addition, self-regulation is a component of temperament; it also influences other aspects of temperament such as activity level and emotionality (Rothbart et al., 2004; Caspi and Shiner, 2006; Rothbart and Bates, 2006). Higher levels of self-regulation for example, would have an effect on children’s behaviors as well as the extent to which they are capable of regulating both the experience and expression of emotion (Wills et al., 1995; Belsky et al., 1998; Posner and Rothbart, 1998; Gerardi-Caulton, 2000; González et al., 2001; Ellis et al., 2004; Eisenberg and Spinrad, 2004; Kim and Kochanska, 2012). Wills et al. (1995) found that high activity level was detrimental to the development of self-regulation. González et al. (2001) found that 7-year-old children scoring high in activity level and impulsivity performed worse on a Stroop task, indicating lower attentional control – a key element of self-regulation. Kim and Kochanska (2012) measured temperament when children were 7 months old and their self-regulation when they were 25 months. They found that negative emotionality (temperament) was negatively correlated with self-regulated compliance to paternal control.

Previous studies have also shown that temperament also influences how self-regulation develops. For example, Duckworth et al. (2013) found that mother ratings of their preschool children’s motor activity predicted their delay of gratification, with higher ratings of motor activity being associated with lower delay time; in addition, shy children, based upon maternal ratings, delayed longer. Mittal et al. (2013) looked at temperament, personality, and parental attachment as correlates of delay of gratification in 2-and 3-year-olds, using the EASI-III and the California Child Q-set. In this study, children were classified into Delay (waited for a larger gift throughout), Touch and Go (waited for a larger gift in the beginning, but could not wait throughout), and Non-Delay (chose not to wait). Results showed that Non-Delayers scored higher on activity than the Touch and Go group. Touch and Go children scored higher on negative emotionality than Delayers.

### The Role of Strategies during Delay of Gratification Tasks

Despite evidence demonstrating that temperament plays a pivotal role in affecting self-regulation, personality is not destiny. How well a child performs in a DoG task is affected not only by children’s temperament and the challenges in the delay situation itself, but also by the strategies children use to cope with those challenges (Peake et al., 2002; Mittal et al., 2013; Neuenschwander and Blair, 2017). In addition to the biological underpinnings (such as temperament) of self-regulation, individual skills developed earlier during social exchanges could also exert a significant influence on self-regulation ability (Luria, 1961). Indeed, over the course of development, children are able to acquire more complex regulatory skills that affect their expression of temperamental characteristics (Rothbart et al., 1994). Thus, how well children perform in a delay of gratification task might be influenced not only by their temperament, but also by skills or strategies emerging from the interaction between temperament and experience.

Past research has indicated that engagement of certain strategies can affect the ability to delay gratification. Mischel and Ebbesen (1970), Mischel et al. (1972), Mischel and Moore (1973), and Mischel and Baker (1975) systematically tested children’s DoG performance using different prompts, such as encouraging children to think about rewards in a non-appetitive manner (e.g., encouraging children to generate their own thoughts that they think are fun), and providing them different contexts, such as removing rewards from view and providing distractions (Mischel and Ebbesen, 1970; Mischel et al., 1972; Mischel and Moore, 1973; Mischel and Baker, 1975; Mischel, 2014). Children who were exposed to both delayed and immediate rewards waited a shorter length of time as compared to children where the reward was hidden (Mischel and Ebbesen, 1970). In addition, children who engaged in self-distraction strategies tended to wait longer. By providing children with external and cognitive distractions from the reward objects, Mischel et al. (1972) found that attentional and cognitive strategies that directed children’s attention away from the rewards facilitated longer waiting. Thus, suggesting distracting “fun” thoughts counteracted the effects of exposure to the rewards, helping children wait longer. Together, these studies highlight the relation between distraction and increased delay time in DoG tasks; however, they do not consider the potential role of individual difference factors such as temperament in this process.

Compared to studies that looked at children’s strategy use during DoG task that were experimentally manipulated by researchers, the amount of studies examined children’s spontaneous use of strategies and how they might affect delay time is relatively small (e.g., Rodriguez et al., 1989; Peake et al., 2002). Rodriguez et al. (1989) examined spontaneous attention deployment strategies and ability to delay gratification in children at risk. They found that directing attention away from the bell or rewards was positively correlated with delay time. Also by analyzing the spontaneous attention deployment used by children during a DoG task, researchers (Peake et al., 2002) have found that, regardless of the instructions to the child – e.g., sit and wait for the delay interval to end or feed the baby bird, reward-focused attention was consistently associated with reduced delay time. In sum, children who were able to distract themselves were able to delay gratification longer.

Although there is a fairly extensive literature on delay of gratification, few studies have explored the interactive effect of children’s temperament and the spontaneous use of strategies in which they may engage or the potential efficacy of those strategies for self-regulatory behavior. In addition, much of the research has focused on early childhood and we know much less about spontaneous strategy use in middle childhood when children have more advanced cognitive skills and knowledge of self-regulatory strategies relevant to delay gratification (Brown and DeLoache, 1978; Mischel and Mischel, 1983; Canton and Kihlstrom, 1987). The current study extends earlier research by assessing, in 7–12-year-old children, the extent to which there are spontaneous strategies that might moderate the effects of temperament on delay of gratification. More specifically, we investigated the extent to which the efficacy of certain spontaneous use strategies on delay time was contingent on particular temperamental variables within this middle childhood age range.

### The Role of Gender

Researchers have demonstrated gender differences in temperament and self-regulation in the early school years (Silverman, 2003; Ready et al., 2005; Else-Quest et al., 2006; Ponitz et al., 2008; Else-Quest, 2012). Maccoby and Jacklin (1974) found that boys tended to have higher activity levels than girls, a difference that increases with age. Boys were also found to be more emotionally volatile than girls, and girls’ negative emotional responses were found to decline more quickly with age than boys’. In a meta-analysis of activity level studies, Eaton and Enns (1986) confirmed that differences in activity level between boys and girls became larger as they aged.

In their meta-analysis of gender differences in temperament in children ages 3 months to 13 years, Else-Quest et al. (2006) grouped all temperament dimensions drawn from three main temperamental theories [those of Buss and Plomin (1975), Thomas and Chess (1977), and Rothbart (1981)] into three broad factors: effortful control, negative affectivity, and surgency. They found consistent gender differences in overall effortful control. Their findings suggested that girls, as compared to boys, were generally better at regulating and managing their attention, inhibiting impulses, and being aware of subtle external changes; by contrast, boys scored higher in overall surgency. There was also a small gender difference in activity level consistent with other findings (i.e., boys higher than girls).

Using the California Child Q-set to rate the personalities of children at 3, 4, 7, and 11 years engaging in a DoG task, Funder et al. (1983) found that the ability to delay gratification was associated with particular personality variables at all four ages. Specifically, they found that boys who were able to delay gratification were more likely to be attentive, to concentrate, and to be reserved, cooperative, and generally impulse controlled than boys who did not delay, who in turn were more likely to be described as irritable, restless and fidgety, aggressive, and generally less self-controlled. Girls who delayed gratification were more likely to be described as intelligent, resourceful, and competent whereas girls who did not delay were more likely to be described as reacting poorly under stress, easily offended, sulky, and whiny. In sum, given this literature, it is possible that gender differences in temperament may mediate gender differences in self-regulation.

### The Purpose of the Current Study

The current study fills gaps in the previous literature on delay of gratification. In particular, we integrate research on temperament and strategy use to determine the relation between temperament dimensions, children’s strategy use, and their ability to delay gratification. Additionally, we examine the important role of gender. The purposes of the current study include (1) investigate relations between temperament dimensions, children’s strategy use, and their ability to delay gratification; (2) ascertain whether spontaneous strategies that early adolescents use during a delay of gratification task *moderate* the effect of temperament on self-regulation; and (3) determine the extent to which childhood temperament *mediates* the relationship between gender and self-regulation. Consistent with past work (e.g., Mischel et al., 1972), we hypothesized that strategies which draw children’s attention away from the reward would be associated with greater delay time. We also hypothesized that higher levels of activity level would be associated with decrease delay time, but that relation would be moderated by effective strategy use. Given that delay of gratification requires self-regulation skills such as inhibitory and impulsive control, and past research has demonstrated that girls score higher on these abilities (e.g., Else-Quest et al., 2006), we hypothesized that girls would have longer delay times than boys. We also hypothesized that activity level would mediate the relationship between gender and delay time.

## Materials and Methods

### Participants

Data were obtained in a longitudinal study, approved by the Cornell University Institutional Review Board, of the role of poverty in children’s development. There were 349 participants (182 boys, 167 girls) who were recruited from rural upstate New York Co-operative Extension and public school districts. The mean age of the children was 9.18 years (*SD* = 1.17). The sample was predominantly Caucasian (90.8%), with approximately one half coming from low-income families. In the present sample, the mean income-to-needs ratio was 1.67 (*SD* = 1.10) with 37.2% of participants living at or below the federal poverty line (defined as income-to-needs ratio equal to 1). Informed parental consent and child assent was obtained for all participants and confidentially was assured. Families were paid for their participation. Only tasks relevant to the current hypotheses are discussed here. Data for these tasks were collected in 1995–1996.

For the current study, 82 participants (23.5%) were excluded from the analyses for the following reasons: broken VCR tapes (delay of gratification task was recorded in VCR tape), blurry scene (coders were not able to detect children’s attention since the video was too dark) and missing gender information. The data from 267 participants (145 boys and 122 girls) were analyzed in our study.

### Procedure

Using a standard protocol, data were collected in participants’ homes by two trained undergraduate research assistants. Temperament was assessed via an interview with the child’s mother while delay of gratification was measured with the child in a separate room.

### Measures

#### Temperament

The parent report of Buss and Plomin’s (1984) EAS Temperament survey was used to assess children’s temperament. The EAS survey consists of 20 items rated on scales from 1 (not typical of their child) to 5 (very characteristic of their child). The EAS survey measures three dimensions of temperament: activity, emotionality, and sociability. Activity represents a child’s general level of energy output. Examples of activity items include: “Child is always on the go”; “Child is very energetic.” Emotionality refers to the intensity of emotional reactions. Examples of emotionality items include: “Child cries easily”; “Child reacts intensely when upset.” Sociability represents a child’s tendency to affiliate and interact with others. Examples of sociability items include: “Child prefers playing with others rather than alone”; “Child finds people more stimulating than anything else” (Buss and Plomin, 1984). The EAS survey has been shown to have good internal consistency for each scale and stability of temperament traits over time (Bould et al., 2013).

#### Delay of Gratification

Prior to the task, children were instructed to sit in front of a plate of candy and a bell. They were told that if they waited until the experimenter returned (after 30 min), they would receive double the amount of candy; however, they could press the bell at any point to terminate the waiting time. They were also told that if they rang the bell, they would receive only the candy in front of them instead of the larger reward that would come if they successfully waited. In order to ensure children understood the procedure, the experimenter left the room, telling the child to ring the bell to signal them to return. After answering any questions and experimenter left the room again, the protocol began and the delay time started. Participants were not told how long they would have to wait for the experimenter to return. The whole task was videotaped. The duration of time they waited was recorded. Children’s behavior during the DoG task was coded for strategy use.

#### Self-regulatory Strategies

The coding of the spontaneous strategies used by the children during the delay of gratification task was adapted from Mauro and Harris (2000), as described next.

Attention Averted: the child appears to be focusing his or her attention on something other than the candy or bell.

Attention to Candy: the child focuses his or her attention on the candy, but does not physically touch it.

Attention to Bell: the child focuses his or her attention on the bell but does not physically touch it.

Manipulating Bell: Touching, feeling, or spinning the bell. Does not include playing with child’s own reflection on the bell.

Imaginative/Symbolic: child appears to be imagining scenarios. Examples include pretending to shoot a gun, playing with hands in a story-like way, and other types of what appeared to be fantasy play.

Imaginative with Bell: child engages with own reflection on the bell; using the bell as an object in what appears to be an imaginative scenario.

Each video was coded in 15-s intervals for the presence of each strategy (1 = strategy showed; 0 = strategy not showed) (Cuskelly et al., 2006). For children who pressed the bell before the end of the 30 min waiting period, coding continued until the child pressed the bell. For each child, a sum score was then computed. Inter–rater reliability was analyzed using the Kappa statistic to insure consistency among three trained coders, who coded a random sample of one out of five videos. For each behavior item, agreement was assessed between each pair of coders. The range of Cohen Kappa was between 0.701 (*p* < 0.001) and 0.926 (*p* < 0.001), showing substantial inter-rater reliability (Landis and Koch, 1977).

Based on previous studies indicating the effectiveness of distraction and attention away strategies in a DoG task (e.g., Mischel and Ebbesen, 1970; Mischel et al., 1972; Rodriguez et al., 1989; Baumeister et al., 1994; Peake et al., 2002), in the current study all strategies that took children’s attention away from the bell and candy were coded as effective strategies and any strategies that brought children’s attention to the bell and/or candy were coded as ineffective. According to this criterion, the following strategies were coded as effective strategies: Attention Averted and Imaginative/Symbolic; these other strategies were coded as ineffective: Attention to Candy, Attention to Bell, Manipulating Bell, and Imaginative with Bell. Besides calculating the total frequencies of effective and ineffective strategies, we also computed ratio values by dividing frequencies of effective and ineffective strategy with total delay time. We tested both strategy proportion scores and total frequencies of effective and ineffective strategy in all of the analyses.

## Results

Temperament was scored according to the guidelines provided by Buss and Plomin (1984). The mean score for emotionality was 2.75 (*SD* = 0.93), the mean activity score was 3.78 (*SD* = 0.82), and the mean sociability score was 3.51 (*SD* = 0.69). **Table 1** provides the mean, standard deviation, and frequency of each strategy. The total effective strategy score was 85.72 (*SD* = 37.31), which was the sum of the means of Attention Averted and Imaginative/Symbolic. The total ineffective score was 44.91 (*SD* = 33.12), which was the sum of the means of Attention to Candy, Attention to Bell, Manipulating Bell, and Imaginative with Bell.

**Table 1:** Descriptive statistics for strategies, overall and separately by gender.

Strategy	*M*	*SD*	Boy		Girl	
			Mean	*SD*	Mean	*SD*
Attention Averted	81.73	35.71	75.02	35.77	89.22	34.29
Attention to Candy	28.99	27.05	30.67	27.87	27.11	26.11
Attention to Bell	13.10	14.50	13.76	15.18	12.37	13.74
Manipulating Bell	1.22	4.31	1.78	5.63	0.58	1.80
Imaginative/symbolic	3.99	9.58	4.75	11.87	3.15	6.04
Imaginative with Bell	1.61	5.71	2.37	7.59	0.75	1.85
Effective Strategies (total frequency)	85.72	37.31	79.76	38.37	92.37	35.08
Effective Strategy (divided by total delay time)	0.91	0.23	0.89	0.26	0.92	0.20
Ineffective Strategies (total frequency)	44.91	33.12	48.58	34.90	40.81	30.67
Ineffective Strategy (divided by total delay time	0.49	0.33	0.55	0.34	0.42	0.31

*Strategies that took children’s attention away from the reward were identified and combined as Effective strategies, which included the following strategies: Attention Averted and Imaginative/Symbolic tactics. Strategies that brought children attention to the bell and candy were identified and combined as Ineffective strategies, which included the following strategies: Attention to Candy, Attention to Bell, Manipulating Bell, and Imaginative with Bell.*

### Temperament and Children’s Strategy Use

Results summarized in **Table 2** summarizes the correlation among strategy use and temperament dimensions. Delay of Gratification, was significantly negatively correlated with activity temperament, *r* = -0.18, *p* = 0.004, was unrelated to emotionality temperament, *r* = -0.02, n.s, and sociability temperament, *r* = -0.05, n.s. In regard to the relationships among general effective strategy, ineffective strategy, and child temperament, we found that both total frequency and ratio of ineffective strategy were significantly negatively correlated with levels of the activity temperament (frequency: *r* = -0.20, *p* = 0.004; ratio: *r* = -0.16, *p* = 0.02) and sociability temperament (frequency: *r* = -0.20, *p* = 0.001; ratio: *r* = -0.26, *p* < 0.01), but unrelated to emotionality. Frequency of effective strategy was unrelated to activity, *r* = -0.02, n.s., sociability, *r* = -0.01, n.s., and emotionality, *r* = 0.07, n.s. Ratio of effective strategy was significantly correlated with activity, *r* = 0.20, *p* < 0.01 and unrelated to emotionality, *r* = -0.08, n.s., and sociability, *r* = -0.06, n.s.

**Table 2:** Correlations among demographic, strategies and temperament variable.

variables	1	2	3	4	5	6	7	8	9	10	11	12	13	14	15	16
(1) Age	–															
(2) Income	0.04	–														
(3) Delay Time	0.16**	0.18**	–													
(4) Attention Averted	0.08	0.16*	0.81**	–												
(5) Attention to Candy	0.19**	0.16*	0.40**	–0.10	–											
(6) Attention to Bell	–0.14*	–0.06	0.18**	0.10	–0.01	–										
(7) Manipulating Bell	–0.04	–0.07	–0.05	–0.12	–0.03	0.26**	–									
(8) Imaginative/Symbolic	0.03	–0.08	0.09	0.04	0.10	0.02	0.14*	–								
(9) Imaginative with Bell	–0.10	–0.11	0.07	–0.07	–0.04	0.57**	0.09	0.08	–							
(10) Effective Strategy (frequency)	0.09	0.13	0.80**	0.97**	–0.07	0.10	–0.08	0.29**	–0.04	–						
(11) Effective Strategy (ratio)	–0.13	–0.12	–0.19**	0.31**	–0.63**	–0.11	–0.12	0.33***	–0.15*	0.38**	–					
(12) Ineffective Strategy (frequency)	0.07	0.08	0.41**	–0.07	0.80**	0.56**	0.23*	0.13	0.40**	–0.03	–0.61**	–				
(13) Ineffective Strategy (ratio)	–0.09	–0.04	–0.23**	–0.55**	0.48**	0.46**	0.31**	0.06	0.33**	–0.51**	–0.45**	0.69**	–			
(14) Emotionality	–0.01	–0.20**	–0.02	–0.07	0.01	0.13	0.02	–0.02	0.08	–0.07	–0.08	0.08	0.06	–		
(15) Activity	–0.08	–0.06	–0.18**	–0.04	–0.25**	0.01	–0.04	0.05	0.03	–0.02	0.20**	–0.20**	–0.16*	–0.04	–	
(16) Sociability	–0.05	0.03	–0.06	0.02	–0.16*	–0.11	–0.03	–0.10	–0.16*	–0.01	0.06	–0.20**	–0.26**	0.10	0.43^∗∗^	–

*Effective Strategy (ratio) = frequency of effective strategy/total delay time; Ineffective strategy (ratio) = frequency of ineffective strategy/total delay time. ^∗^*p* < 0.05, ^∗∗^*p* < 0.01.*

### Does Strategy Use Moderate the Relationship between Temperament and Delay of Gratification?

In the next set of analyses, we investigated whether the relationship between child activity level and delay of gratification ability was moderated by the strategies the children used during the DoG task. We focused only on activity level, as it was the only temperament dimension correlated with delay time. (For completeness, we did run regression analyses for the other two dimensions, but found no significant relations with delay time, and no significant interaction effects with strategy use). We first tested the moderating roles of frequencies of both effective and ineffective strategies between activity temperament and delay time, and then assessed the moderating roles of ratio values of effective and ineffective strategy between this relationship.

**FIGURE 1:**
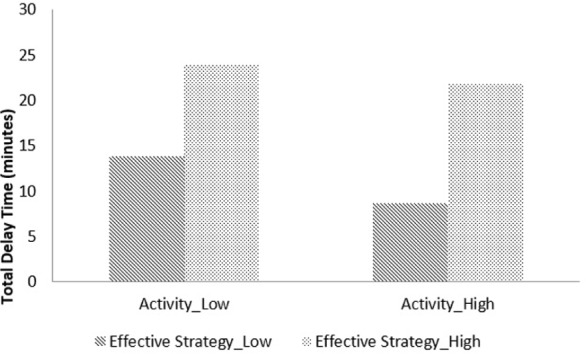
Moderation analysis with activity temperament as predictor, effective strategy as moderator, and total delay as outcome. Both activity level and effective strategy use were plotted at plus/minus one *SD* from their mean.

We conducted moderation analyses and probed the interaction slopes using PROCESS macro developed for SPSS (on a 5000 bootstrapping) (Hayes, 2013). According to the correlation results (**Table 2**), delay time was significantly correlated with family income, *r* = 0.18, *p* = 0.003, and child’s age, *r* = 0.16, *p* = 0.01. Thus, our models controlled for participants’ age, gender, and family income. The results indicated that the overall model was significant, *F*(2,209) = 52.72, *p* < 0.001, *R*^2^ = 0.70. **Table 3** illustrates the results. There was a main effect of activity level, *b* = -1.28, *SE* = 0.40, 95% CI [-2.07, -0.50], frequency of effective strategies, *b* = 0.18, *SE* = 0.01, *p* < 0.001, 95% CI [0.15, 0.20], and income, *b* = 0.81, *SE* = 0.33, 95% CI [0.16, 1.47]. The interaction term (activity level by effective strategies) was also significant, *b* = 0.05, *SE* = 0.02, *p* = 0.005, 95% CI [0.02, 0.08]. As shown in **Figure 1**, regarding the conditional effects of activity level on DoG at plus/minus one *SD* from mean, results suggested there was no relationship between activity level and DoG for children who score high on effective strategy use. Activity level was associated with DoG for children who used a low frequency of effective strategies, *b* = -3.12, *SE* = 0.96, *p* < 0.001, or an average amount, *b* = -1.29, *SE* = 0.39, *p* = 0.002. Note the data in **Figure 1** were plotted at ±1 SD for descriptive purposes only. The inferential interaction analysis maintained the continuous nature of the variables. Interestingly, ineffective strategies use was also positively correlated with delay time, *r* = 0.41, *p* < 0.01. We then ran another set of analyses to examine whether ineffective strategy use moderated the relationship between activity level temperament and delay time; it did not.

**Table 3:** Interaction between temperament and effective strategies use in predicting delay time.

	Delay time			
Variable	*B*	*SE*	*t*	95% CI
Age	0.54	0.29	1.89	[-0.02, 1.10]
Gender	–0.25	0.71	–0.34	[0.71, -0.34]
Income	0.81ˆ*	0.33	2.47	[0.33, 2.47]
Effective Strategies	0.18ˆ***	0.01	12.43	[0.01, 12.43]
Activity Level	–1.29ˆ**	0.40	–3.23	[0.40, -3.23]
Effective Strategies ^∗^ Activity Level	0.05ˆ**	0.02	2.84	[0.02, 2.84]

*^∗^*p* < 0.05, ^∗∗^*p* < 0.01, ^∗∗∗^*p* < 0.001.*

Next, we used the same method to test the moderating roles of ratios of effective and ineffective strategy between activity temperament and delay time. The results indicated that both ratios of effective and ineffective strategy did not significantly interact with activity temperament in predicting delay time.

### Are There Gender Differences in Temperament and Delay of Gratification?

**Table 4** provides the means and standard deviations for temperament and delay of gratification scores separately by gender.

**Table 4:** Descriptive statistics for temperament and delay time separately by gender.

	Boy		Girl	
	Mean	*SD*	Mean	*SD*
Emotionality	2.71	0.89	2.79	0.98
Activity	3.92	0.77	3.59	0.85
Sociability	3.45	0.66	3.59	0.73
Delay time	22.57	9.59	25.47	8.02

ANCOVA analysis was used to assess any gender differences in delay time and temperament (controlling for age, and family income). The ANCOVA, *F*(1,259) = 6.42, *p* = 0.012, η^2^ = 0.02, demonstrated that girls (*M* = 25.47, *SD* = 8.02) had significantly longer delay times than boys (*M* = 22.57, *SD* = 9.59).

Regarding temperament, the analyses showed that the only significant gender difference was on activity level, on which boys (*M* = 3.92, *SD* = 0.77) scored significantly higher than girls (*M* = 3.59, *SD* = 0.85), *F*(1,276) = 13.01, *p* < 0.001, η^2^ = 0.05. The gender differences on the emotionality variable (Girls: *M* = 2.78, *SD* = 0.98; Boys: *M* = 2.70, *SD* = 0.88) and the sociability variable (Girls: *M* = 3.58, *SD* = 0.73; Boys: *M* = 3.44, *SD* = 0.66) were not statistically significant.

### Does Activity Level Mediate the Relationship between Gender and Delay Time?

In regard to the relationship between temperament and delay of gratification, Pearson correlational analysis (**Table 2**) indicated that only child’s activity temperament was significantly negatively correlated with delay time, *r* = -0.18, *p* = 0.004. No statistically significant association was found between the other two temperament dimensions (Emotionality and Sociability) and delay time.

**FIGURE 2:**
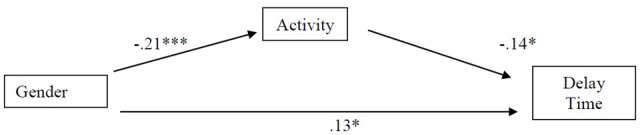
Results of the regression analysis show that the effect of Gender on Delay time is mediated by Activity temperament. The numbers are standardized regression coefficients. ^∗^*p* < 0.05, ^∗∗^*p* < 0.01, ^∗∗∗^*p* < 0.001.

To determine whether activity temperament has a mediation effect between gender and delay time, we followed the recommendations of Preacher and Hayes (2004), using bootstrapping procedures to compute a 95% confidence interval around the indirect effect (e.g., the path through the mediator). We statistically controlled for child’s age and family income. With gender as the independent variable, activity level as the mediator, and delay time as the dependent variable, analyses (**Figure 2**) revealed that the indirect effect via activity equaled 0.57, the 95% confidence interval ranging from 0.12 to 1.42, indicating a significant mediation effect.

## Discussion

Previous literature makes it clear that the ability to delay gratification is an important component of self-regulation abilities that foreshadow important aspects of human development, including adaptation and coping, social interaction, and academic achievement (Eisenberg and Fabes, 1992; Eisenberg et al., 1993; Wills et al., 2006; Vazsonyi and Belliston, 2007; Mischel, 2014). Results from the present study were consistent with previous findings indicating that ability to delay gratification is related to a child’s temperament (e.g., Mittal et al., 2013) and gender (e.g., Funder et al., 1983; Else-Quest et al., 2006). We also demonstrate that gender differences in temperament, namely activity level mediates the gender differences in the ability to delay gratification. Moreover, by systemically examined children’s spontaneous strategy use during delay of gratification task, we found that effective strategy use, which enables better self-regulation in the DoG task, moderates the relation between activity temperament and self-regulation. Our findings are important in that they raise the issue that interventions aimed at improving children’s self-regulation should consider the importance of individual differences such as temperament.

### The Interaction between Temperament and Strategy Use on Delay Time

Previous studies had found that both the experimentally primed distraction strategies and spontaneous attention deployment increase delay of gratification in a waiting paradigm. In their early study, Mischel and Ebbesen (1970, p. 335) suggested that one simple and effective delay strategy was “converting the aversive waiting situation into a more pleasant non-waiting one.” They found that when children were avoid looking at the reward, engaging themselves doing something else, such as talking to themselves, singing songs, or inventing games with their hands and feet, they were more able to delay. Additionally, the negative effect of high activity level on delay time has been consistently demonstrated (Mischel and Ebbesen, 1970; Baumeister et al., 1994; Mittal et al., 2013). Consistent with this work, we found that when the children used more effective strategies, the relation between activity level was not associated with performance in the DoG task. However, when they used less effective strategies, their activity temperament showed a significant negative influence on their delay time.

One possible interpretation of why using effective strategies helped children with higher activity level delay longer in DoG task is that since children higher in activity temperament react poorly to enforced idleness, when they are able to direct their attention away from the situation of enforced idleness and toward something else in the room or use their imagination to perform fantasy play, their tendency to be restless and energetic is temporarily subdued by attention control. Thus, these strategies helped children with higher activity temperament distract themselves away from the enforced idleness of the task and delay longer in the task.

One additional important consideration is the conceptual relations between temperament and self-regulation. In our model, we presuppose that temperament dimensions such as emotionality, activity level and sociability tap into basic motoric, approach/avoid biological tendencies that are present at birth (Calkins, 2007). One major mile stone is to develop the self-regulatory capacities to modulate this reactivity. However, it is unclear whether self-regulatory abilities may in essence be integral to temperament. For example, is it the case that children who are low on activity level inherently have higher levels of self-regulatory capacities (for a discussion see Bridgett et al., 2013 and Nigg, 2016). Disentangling these aspects and their relations to outcomes is important for future research.

In terms of ineffective strategies, we did not find a moderating effect between temperament and delay time. Interesting in our data, we found that strategies which has been delineated as “ineffective” (frequency) were positively correlated with total delay time. This suggests that perhaps any strategy use, even those that are deemed “ineffective” may be beneficial to a certain extent in helping children to delay. One interpretation of this is simply that any strategy which children are using may be better than nothing. However, it is important to note that in our data, only effective strategies interacted with children’s temperament in predicting total delay time. In addition, the correlation results showed that ineffective strategy use was negatively correlated with activity temperament. One possible reason for this is that children high on activity levels may have difficulty with attention, and staying still, thus they may be less likely to engage in secondary strategies.

Interestingly, although the frequency of effective strategy moderated the relationship between activity temperament and delay time, the ratio of effective and ineffective strategy did not. That means, how fast children use effective strategy or ineffective strategy did not interact with their temperament in predicting delay time. As both ratios of effective and ineffective strategy were negatively correlated with delay time, it is possible that using strategies too quickly actually prevent children wait longer. Thus, it is possible that children who are able to use effective strategy to down-regulate their arousal when they felt anxious in DoG task, could wait longer; and they more strategy they use, the longer they can wait. While using strategy too quickly did not help them wait longer.

Additionally, we found that family income was positively correlated with delay time. Children who came from a higher income family were able to delay longer in this task. This result was consistent with previous studies that low-income children have multiple self-regulatory deficits and exhibited weaker self-control (e.g., Blair and Raver, 2012; Evans and Kim, 2013). For completeness sake, we tested three way interactions with income, temperament, and strategy use and did not find that the moderating effect of strategy use was influenced by income. Future, research should, however, considered more thoroughly the extent to which income may influence the type of strategy as well as their relative effectiveness.

### Temperament and Gender

Results from our study showed that compared to girls, boys had greater difficulties with self-regulation as shown by significantly lower delay time. Boys also scored significantly higher on activity temperament. Both of these findings are consistent with previous findings (Maccoby and Jacklin, 1974; Eaton and Enns, 1986; Else-Quest et al., 2006). Results also showed that children with higher activity temperament were less able to delay overall, which is consistent with findings by Else-Quest et al. (2006), Mittal et al. (2013), and Duckworth et al. (2013). Since most previous research focused on younger children, our findings extend these prior studies on gender and temperament in relation to delay of gratification to older children. Additional mediational analyses show that these gender differences in DoG are mediated, at least in part, by the higher activity temperament levels of males. Buss and Plomin (1984, p. 94) described children with higher activity temperament as showing a reaction to enforced idleness, in which they seek outlets for their energy and become more restless during idle periods, such as during a DoG task. In their meta-analysis of temperament, Else-Quest et al. (2006) showed that boys scored higher overall on the broad factor surgency, which included activity. They also showed that girls scored higher on the broad factor effortful control, which included attention. Taken together with our findings, it is possible that the reason males had poorer performance at a self-regulation task may be due to boys’ consistently scoring higher in activity and its related temperamental dimensions, and the finding that males scored lower in effortful control and the associated temperamental dimensions. Thus, gender differences in activity temperament are able to explain at least part of why boys are less likely to succeed at the DoG task specifically. Extending beyond our data, this mediational pattern of gender differences in temperament might also help explain why boys in general are poorer in self-regulatory functioning than girls.

### Implications

Results from this study suggest that children’s temperamental activity level (which appears early in life and is relatively stable) is directly related to their ability to delay gratification, the strategies they employ, and the effectiveness of those strategies. The ability to delay gratification has been shown to be crucial to positive growth; thus, it is important to understand what kind of factors may contribute to individual differences in this ability. High temperamental activity level has been shown in both this study and a few other recent studies to have a negative effect on delay of gratification (e.g., Duckworth et al., 2013; Mittal et al., 2013). However, strategy use was found to moderate the effects of high activity level temperament on self-regulatory behavior. Children with high activity temperament benefitted from greater use of effective attentional strategies that provided a means to divert attention away from the object of immediate gratification (large plate of candy herein). Children low in activity temperament did not gain much advantage from use of these attentional avoidance strategies. Future research should focus on which strategies may be helpful to children with specific temperaments, particularly children with a temperament characterized by high activity levels. Perhaps instructing children who have high activity temperament with effective strategies may help them improve in the DoG task. Another future priority would be investigation of which strategies, if any, might be effectively employed in the delay of gratification task for children low in activity temperament.

## Ethics Statement

The study was carried out in accordance with the recommendations of “APA ethical guidelines” with written informed consent from all subjects. All subjects gave written informed consent in accordance with the Declaration of Helsinki. The protocol was approved by the “Cornell University Institutional Review Board.”

## Author Contributions

FH, SD, and GE conceived and designed the study. FH conducted the analysis. FH and AL drafted the paper. SD and GE provided critical feedback.

## Conflict of Interest Statement

The authors declare that the research was conducted in the absence of any commercial or financial relationships that could be construed as a potential conflict of interest.
